# Harvest Timing of Standing Corn Using Near-Infrared Reflectance Spectroscopy

**DOI:** 10.3390/s24051397

**Published:** 2024-02-21

**Authors:** Matthew F. Digman, Jerry H. Cherney, Debbie J. R. Cherney

**Affiliations:** 1 Department of Biological Systems Engineering, University of Wisconsin-Madison, Madison, WI 53706, USA; 2 Section of Soil and Crop Sciences, School of Integrative Plant Science, Cornell University, Ithaca, NY 14853, USA; jhc5@cornell.edu (J.H.C.); djc6@cornell.edu (D.J.R.C.)

**Keywords:** *Zea mays*, corn silage, whole-plant corn silage, forage moisture

## Abstract

Harvesting corn at the proper maturity is important for managing its nutritive value as livestock feed. Standing whole-plant moisture content is commonly utilized as a surrogate for corn maturity. However, sampling whole plants is time consuming and requires equipment not commonly found on farms. This study evaluated three methods of estimating standing moisture content. The most convenient and accurate approach involved predicting ear moisture using handheld near-infrared reflectance spectrometers and applying a previously established relationship to estimate whole-plant moisture from the ear moisture. The ear moisture model was developed using a partial least squares regression model in the 2021 growing season utilizing reference data from 610 corn plants. Ear moisture contents ranged from 26 to 80 %w.b., corresponding to a whole-plant moisture range of 55 to 81 %w.b. The model was evaluated with a validation dataset of 330 plants collected in a subsequent growing year. The model could predict whole-plant moisture in 2022 plants with a standard error of prediction of 2.7 and an R^2^P of 0.88. Additionally, the transfer of calibrations between three spectrometers was evaluated. This revealed significant spectrometer-to-spectrometer differences that could be mitigated by including more than one spectrometer in the calibration dataset. While this result shows promise for the method, further work should be conducted to establish calibration stability in a larger geographical region.

## 1. Introduction

Whole-plant corn silage is the primary forage in dairy cow diets, with over 90% of farms nationwide incorporating it into their rations [1]. Ref. [2] recorded inclusion rates of corn silage ranging from 40 to 60% of ration mass among Wisconsin dairy herds. The broad acceptance of corn silage is attributed to its ability to be produced on-farm, high energy content, and economical production costs.

Harvest timing must be properly managed to realize the full potential of corn silage. Early harvests yield highly digestible, high-moisture corn but compromise grain yield, risk poor anaerobic fermentation, and may lead to effluent production [1]. Conversely, late harvests result in higher grain content at the expense of fiber digestibility, fermentation extent, and aerobic stability. Therefore, moisture content, often used as a surrogate for corn maturity, is a key indicator of anaerobic fermentation and the long-term preservation of corn silage.

The kernel milk line has long been used as a reliable method for estimating corn forage dry matter (DM) and the time to harvest [2]. However, new varieties of corn, such as waxy, leafy, brown midrib, etc., and different storage types (bunker, silo, bag, etc.) have changed the relationship between the kernel milk line and desired DM. Several other options are available for the on-farm monitoring of corn moisture. These include selecting a representative set of plants, drying a chopped subsample, or utilizing a NIR-equipped harvester to chop a short pass in the field [3,4]. Yet, each method requires a chopped, whole-plant sample for moisture assessment.

Ear sampling would be an improvement over collecting whole plants as less material would be collected from the field, and the ear could be dried without chopping. Our previous research revealed a good correlation (R^2^ = 0.89) between ear and whole-plant corn moisture content over a two-year study [5]. Nevertheless, collecting and drying corn ears is labor intensive, constraining spatial and temporal sampling. If corn ears could be evaluated for moisture in situ, they could be left on the plant, enabling more extensive sampling. While corn grain moisture testers using dielectric measurements have been on the market for some time, they are not reliable for predicting grain moisture greater than 45 % w.b. [6]. However, near-infrared (NIR) spectrometers have been advertised for this specific application [7].

Our previous work on predicting ear moisture in situ employed a handheld NIR spectrometer that measured light reflectance in the 740–1070 nm range [6]. A calibration was developed to predict corn ear moisture using this device. While the method produced a high correlation between predicted and oven-dry moisture (R^2^ = 0.92), the standard error of prediction was high (SEP = 3.6), and a significant instrument-to-instrument bias was observed [5]. It is possible that the inclusion of just one known absorbance band for water (970 nm, O-H overtone) could have been a key limitation in that work [8].

Many handheld NIR instruments incorporate a broader wavelength range, including water absorbance bands at 970 nm, 1450 nm, and 1950 nm [9]. This extended wavelength range could capture additional information and improve the prediction of moisture content. Therefore, an enhancement to our previous work would involve the examination of an instrument with a wider scanning range, potentially leading to more precise moisture predictions and reducing the instrument-to-instrument bias.

In this study, we evaluated three different approaches to predicting standing corn dry matter (whole-plant moisture). The first approach employed a previously established correlation between oven-dried ear moisture and whole-plant moisture to predict whole-plant moisture.

The second approach used the same ear-to-whole-plant moisture relationship, but instead of oven-dried measurements, it utilized spectra from handheld NIRS technology to predict moisture content. This method explored the effectiveness of a handheld NIRS device in predicting ear moisture, which could then estimate whole-plant moisture.

The third approach directly predicted the whole plant’s moisture content using handheld NIRS scans of corn ears, thus examining whether a handheld NIRS device can estimate whole-plant moisture directly, bypassing the need for separate ear moisture prediction.

## 2. Materials and Methods

### 2.1. Field Sampling

In the fall of 2021, fifteen plants per field were collected from forty corn fields in central, western, and northern New York state and were randomly selected for sampling. In the fall of 2022, five plants per field were collected from sixty fields. Within each field, plants were cut at a 20 cm stubble height for analysis. The sampling process avoided selecting plants from outside rows, as these plants often have two fully developed ears due to reduced competition and increased light exposure.

Before scanning, the husks were removed from the ears and included with the rest of the stover. The ears were scanned three times from top to bottom while rotating the ear using each handheld NIRS device employed in the study. In 2021, the sampling period ranged from 27 August to 5 October, with a total of 610 plants sampled, and in 2022, sampling commenced on 25 August and concluded on 1 October, for a total of 330 plants.

After the NIR scanning process, the corn ears were detached from the stalks while keeping them intact. Subsequently, they were dried in a forced-air oven at 60 °C for at least five days or until a constant weight was achieved. Corn stover, excluding the grain and cob, was chopped using a Hege 44 stationary plot chopper (Wintersteiger Inc., Salt Lake City, UT, USA). The chopped stover was then dried at 60 °C for two days.

The ears were scanned using three handheld NIRS devices of the same model, specifically the NeoSpectra-Scanner manufactured by Si-Ware Systems Inc. based in Cairo, Egypt. The NeoSpectra-Scanner is a compact device that integrates a rechargeable battery for power, a light source, light collection optics, a monolithic Michelson interferometer, an uncooled InGaAs photodetector, system control and data processing electronics, as well as Bluetooth connectivity.

The device is equipped with a 10 mm collection window, and the accompanying software, NeoSpectra Scan software V1.0, allows for data collection and processing. The software reports spectra within the 1350–2550 nm range, with a variable step size between 2.5 and 8.8 nm and a wavelength resolution of 16 nm. Data were collected on the instrument and transferred to NeoSpectra Scan. Each ear was scanned three times using the device, and the resulting scans were averaged to obtain a representative spectrum for each ear.

### 2.2. Calibration Development

Partial least squares regression (PLS) was used to explore the relationship between response (ear moisture or whole plant moisture) and predictor variables (spectra). Data management and analysis, including autoscaling, mean centering, math pretreatment, regression, cross-validation, and the computation of model performance statistics, were performed using MATLAB (R2020b, MathWorks, Inc., Natick, MA, USA) and PLS_Toolbox (R9.0, Eigenvector Research, Inc., Manson, WA, USA).

In all the calibration models, the spectra from the three repeated scans were averaged and converted to absorbance (log R^−1^). Spectral preprocessing was explored using the *model optimizer* function within PLS_Toolbox. The methods considered were none, autoscaling, multiplicative scatter correction (MSC), and the Savitzky–Golay derivative (SGD). The derivative treatment was further optimized by surveying the process parameters, including the derivative order of first or second, the window width of 9–27 variables, and the polynomial order of two or three. The impact of pretreatment on the number of latent variables, the ratio of the standard error of calibration to cross-validation, and the coefficient of determination were considered before selecting the optimal spectral transformation.

The number of latent variables (LVs) was selected by plotting the variance explained by each LV for both the cross-validation and calibration training sets. The optimal number of LVs was selected at the first local minimum, given that the root mean standard error of calibration (RMSEC) was not divergent from the root mean standard error of cross-validation (RMSECV). Cross-validation utilized the Venetian blinds method with ten splits and a blind thickness of one. The root mean standard error of the calibration, validation, and prediction was computed as RMSE (C, V, P) = $\sqrt{\frac{1}{N}\sum_{i=1}^{N}{\left(P_{i}-L_{i}\right)}^{2}}$, where L = laboratory reference, P = NIRS predicted value, and N = the number of samples [10].

The success of each prediction model was assessed with an independent validation set of 330 observations collected in the 2022 harvest year. The model performance was reported based on the coefficient of determination (R^2^P), the root mean square error of prediction (RMSEP), the standard error of prediction (SEP = RMSEP corrected for bias), and the ratio of prediction to deviation (RPD = validation dataset standard deviation SD/SEP). Although this metric can be derived from R^2^ ($RPD=1/\sqrt{1-R^{2}}$), previous researchers have established NIRS calibration performance classifications based on this metric [11]. The following criteria define functional ranges for RPD values: not recommended (0 < RPD < 1.9), rough screening (2.0 < RPD < 2.4), screening (2.5 < RPD < 2.9), quality control (3.0 < RPD < 3.4), process control (3.5 < 4.0), and any application (RPD > 4.0).

## 3. Results and Discussion

### 3.1. Method 1

The initial method employed to predict the moisture content of standing or whole-plant corn involved two steps. First, ear moisture content was predicted using NIRS. Then, this prediction was utilized in conjunction with a previously developed model that established a relationship between ear moisture and whole-plant moisture. In our previous study [5], we successfully demonstrated a quadratic model that accounted for 89% of the variation in ear moisture (EM) when compared to whole-plant moisture (WP):

EM = 0.028WP^2^ − 2.24WP + 78.3 (1)

To assess the effectiveness of this model using our new dataset, we applied Equation (1) to estimate the whole-plant moisture. The validation set consisted of ear moisture data collected in 2022. It is important to note that we purposely excluded the 2021 data for this analysis, as we intended to utilize it for NIRS calibration.

**Figure 1:**
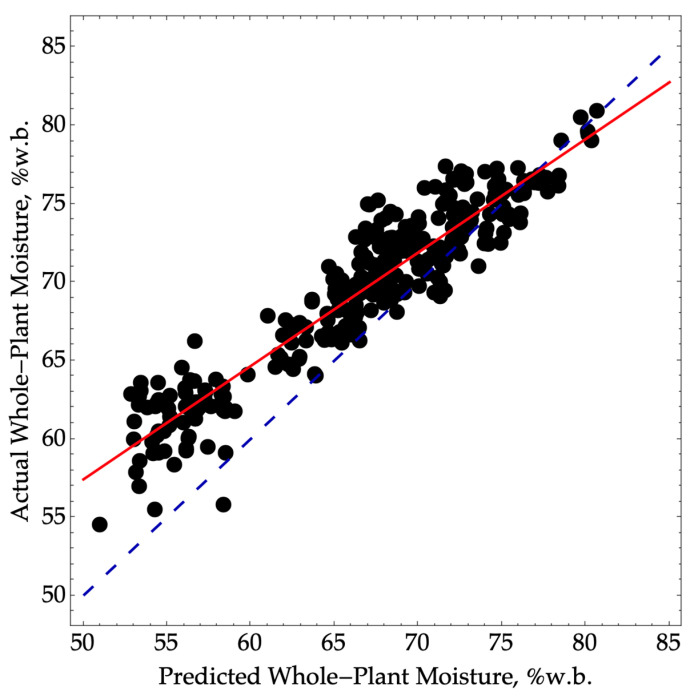
Predicted versus actual whole-plant moisture where oven-dry ear moisture content was used to predict whole-plant moisture using Equation (1). The validation data were collected in the 2022 season (N = 330, RMSEP = 3.9, SEP = 2.7, R^2^P = 0.88) over a range of moisture from 55 to 81. The dashed blue line represents a 1:1 agreement between actual and predicted values. The solid red line corresponds to a linear model between the actual and predicted values.

A total of 330 plants were examined, and we achieved a root-mean standard error of prediction (RMSEP) of 3.9 moisture percentage points, along with an R^2^P value of 0.88 (Figure 1). Although it is possible to account for the bias of 2.8 and the slope of 0.72 through a multi-point calibration process, the precision error cannot be corrected.

According to the performance criteria established by [11], our model for predicting whole-plant moisture from ear moisture would fall into the screening performance category with an RPD value of 2.9. However, it would be on the threshold of the quality control category, as the RPD value (2.9) is close to the cutoff of 3.0.

This result shows promise for determining the optimal harvest timing. However, it assumes that the moisture content of the ear is known. One approach for determining the ear moisture content is to harvest ears manually, weigh them, and dry them in an oven overnight. However, this method requires infrastructure that may not be available on many farms. Another option is to involve a consultant or a forage testing laboratory to carry out the drying process, which would delay the time between sampling and harvest decision making.

### 3.2. Method 2

Predicting ear moisture using a handheld device like the one studied in this research would be desirable to address this limitation and make the process more convenient. However, it is important to consider the moisture prediction error when utilizing the handheld NIRS device for whole-plant moisture prediction. Therefore, the next phase of this study focused on evaluating the performance of the handheld NIRS device in predicting ear moisture.

Since our previous work did not include the same NIRS instrument, we could not simply use the 2021 and 2022 data to evaluate the performance of predicting ear moisture. Therefore, we used the 2021 data to build a model and the 2022 data to evaluate the model performance. In the 2021 dataset, 610 ears were scanned using three instruments, each conducting three scans per ear. Consequently, there were a total of 5481 scans recorded. For this analysis, the scan data were averaged for each ear, resulting in a calibration dataset of 1827 scans.

In 2021, the corn ear moisture content ranged from 26 to 75 %w.b. with an average moisture content of 49 %w.b. and standard deviation of 9.6 moisture percentage points (Table 1). In 2022, 330 ears were scanned with a moisture range of 37–80 %w.b., an average of 55% w.b., and a standard deviation of 10 moisture percentage points (Table 2). Both datasets agree with our previous observations, where the range, average, and standard deviation were 27–81 %w.b., 55 %w.b., and 11 percentage points, respectively [5]. Consequently, a calibration developed with data from any year would include the moisture range and variability expected between growing seasons.

The best-performing calibration (SEC = 2.8, R^2^C = 0.92) in predicting ear moisture content from spectra utilized a second derivative with a smoothing kernel length of 11 and using quadratic interpolation (Table 2, All column). Additionally, there is good agreement between the standard error of calibration and cross-validation (SECV = 2.8), indicating a robust model that is not overfitted. A SEC of 2.8 is numerically better than the 3.1 observed in our previous study when predicting ear moisture with a handheld NIRS in the wavelength range of 740 to 1050 nm [5]. However, this numerical difference is unlikely to have practical implications when selecting an instrument or wavelength range for predicting ear moisture.

We also developed single- and multiple-instrument prediction models to determine whether the prediction model was affected by variability introduced by different instruments of the same make and model. A common strategy for mitigating instrument-to-instrument variability involves building a calibration model using multiple instruments. This approach ensures model stability as additional instruments are deployed in the field. Thus far, the results take this approach.

To evaluate the necessity of spectra from multiple instruments on calibration data, we developed a calibration with each individual instrument and with each possible combination of instruments (Table 1). We subsequently used the 2022 season dataset to evaluate these models (Table 2). Our analysis revealed that predicting the validation set with a single instrument model resulted in an increased RMSEP (see columns labeled 1→ 2, 1→3, 2→1, 2→3, 3→1, 3→2 in Table 2), primarily due to the introduction of a more substantial bias. The bias was lower when the same instrument was used across both seasons (columns 1→1, 2→2, 3→3), indicating that hardware can introduce bias in addition to seasonal variations. Instrument three exhibited the highest inter-seasonal bias of 2.3 moisture percentage points, while instrument one had the lowest at 0.33.

Generally, the slope remained stable regardless of the instruments used in the calibration development. However, instrument three showed numerically lower sensitivity when used as the sole calibration instrument.

The RMSEP was lowest when all instruments were used to develop a calibration to predict the spectra from one instrument in the second season and highest for single-instrument calibrations. The combination of two instruments to predict a third yielded results like the “All” instrument calibration when instrument three was part of the calibration, with RMSEPs ranging from 2.7 to 2.8 moisture percentage points for the 2&3 and 3&1 calibration models. However, the 1&2 model resulted in an RMSEP of 4.9, providing further evidence that instrument three introduced variability to the calibration model that instruments 1 and 2 did not.

Based on these results, we conclude that instrument variability significantly influenced our results and that using two instruments might be insufficient for capturing this variability adequately. This result supports previous work on handheld NIRS devices [12]. Practitioners should consider this instrumental variability in calibration model development for more accurate and reliable predictions. Multi-instrument calibrations or calibration transfer methods should be considered when managing calibrations across multiple instruments.

Typically, plotting predicted ear moisture against actual ear moisture at this stage is customary. However, this study primarily focuses on utilizing these predictions to estimate whole-plant moisture, as this parameter is relevant to the ensiling process and the production of high-quality corn silage. Consequently, our next step involved utilizing the ear moisture predictions obtained from the NIRS as input for Equation (1) to predict the whole-plant moisture content.

### 3.3. Method 3

**Figure 2:**
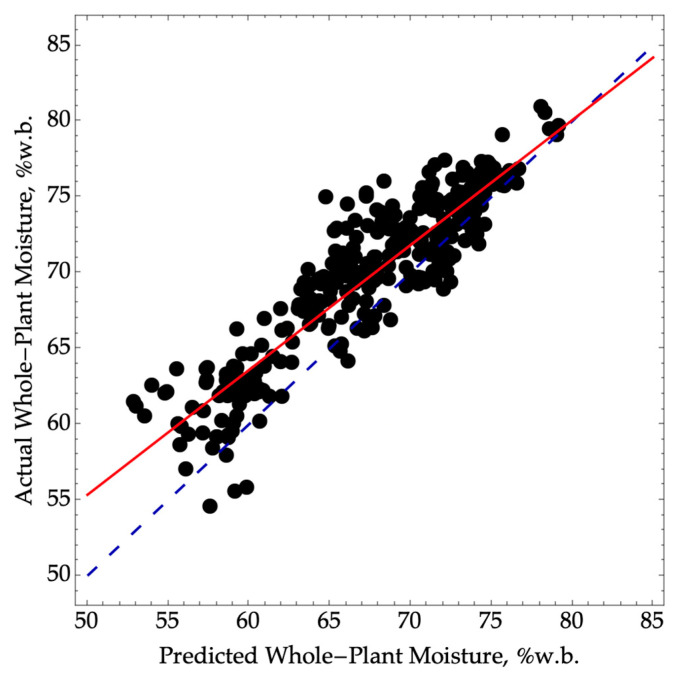
Predicted versus actual whole-plant moisture where NIRS was first used to predict ear moisture and then converted to whole-plant moisture using Equation (1). The validation data were collected in the 2022 season (N = 330, RMSEP = 3.4, SEP = 2.5, R^2^P = 0.82) over a range of moisture from 55 to 81. The dashed blue line represents a 1:1 agreement between actual and predicted values. The solid red line corresponds to a linear model between the actual and predicted values.

When predicting whole-plant moisture by first predicting ear moisture using NIRS and then applying Equation (1), we observed a significant bias, leading to an RMSEP of 3.4 (Figure 2). Additionally, there was a loss of precision with an R^2^P value of 0.82 compared to the oven-dry ear moisture reference method. However, it is worth noting that if we assume a single-point calibration could be conducted each season, achieving a lower SEP of 2.5 would be possible. Despite this improvement, the reduced precision firmly categorizes the model in the screening class.

The final approach we evaluated involved directly predicting the whole-plant corn moisture content using handheld NIRS ear scans. If successful, this approach would eliminate the need for a separate calibration model to predict whole-plant moisture from ear moisture (Equation (1)) and simplify the prediction process. Only one model would be required instead of maintaining two models for ear and whole-plant moisture.

We hypothesized that the spectra obtained from the ear scans would contain information about the physiological state of the corn beyond just grain moisture content. Specifically, we expected the spectra to be sensitive to starch accumulation from sucrose as the ear progresses from the dough to the dent stage of physiological maturity. Both starch and sucrose can influence the spectra captured in the NIRS region.

**Figure 3:**
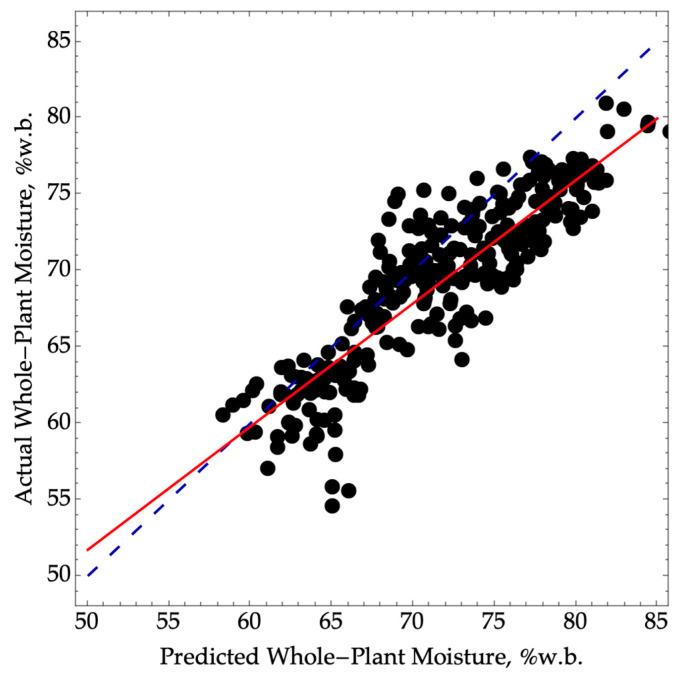
Predicted versus actual whole-plant moisture where NIRS was used to directly predict whole-plant moisture using Equation (1). The validation data were collected in the 2022 season (N= 330, RMSEP = 3.7, SEP = 2.7, R^2^P= 0.79) over a range of moisture from 55 to 81. The dashed blue line represents a 1:1 agreement between actual and predicted values. The solid red line corresponds to a linear model between the actual and predicted values.

However, this direct approach of predicting whole-plant moisture using ear scans resulted in a larger bias and a decrease in the precision of whole-plant moisture prediction, with an R^2^P value of 0.79 (Figure 3). Despite this performance degradation, the model classification would still fall within the screening category with an RPD value of 2.2, like our previous approaches.

Previous methods used for on-farm moisture prediction serve as performance benchmarks for our study despite their requirement for a chopped sample and thus being less convenient. The first group of methods saves time by only partially drying the forage but uses a gravimetric approach and thus has the best agreement with the oven-drying but at considerable savings in time [3,13,14]. Ref. [13] reported that moisture determined with a microwave oven or the Koster method took between 30 and 90 min, depending on the crop species, with corn silage having the longest drying time.

More recently, Ref. [14] compared gravimetric methods to a handheld NIRS for predicting dry matter (DM) content in ensiled forage. They did not report the wavelength range of the instrument but observed that the manufacturer provided the calibration to have a significant bias. After correcting the bias, the instrument performed better in alfalfa silages than in corn. However, their dataset had a sixteen-point dry matter percentage point range in alfalfa compared to an eight-point range in corn, which may have influenced the results.

Another major difference between our work and [14] was that we were able to establish and assess our calibration over a broader range of moisture contents, specifically from 37 to 80 %w.b., in contrast to the narrower range of 57 to 68 %w.b. This is because we were sampling standing corn where they were sampling ensiled corn where the moisture range was limited by harvest management. We also did not observe a large bias when predicting our validation set, as they reported. However, our data were collected in the same region between two growing years, using the same spectrometers and laboratory reference methods.

## 4. Conclusions

This work demonstrated that standing corn dry-matter estimations could be assessed with accurate and precise ear moisture predictions approaching the quality control classification. This was accomplished by applying a previously developed ear moisture model to predict whole-plant corn moisture content for two growing seasons after the model was developed. Furthermore, we demonstrated the practicality of using ear moisture predictions from a handheld NIRS device to estimate whole-plant moisture. This approach exhibited a minimal loss in precision and accuracy compared to using the oven reference to predict whole-plant moisture. Additionally, the study explored the direct prediction of whole-plant moisture using handheld NIRS ear scans. While this method achieved a successful prediction on an independent validation set, it reduced precision and accuracy. However, the performance categorization of screening remained the same. In conclusion, the methodologies presented in this work can be applied to develop a tool for rapidly assessing standing corn moisture content. This tool could be useful in guiding harvest timing decisions for optimizing the production of corn silage.

## Figures and Tables

**Table 1:** Statistics of calibration results for a calibration developed from 2021 ear scans with three handheld instruments, which were utilized to develop a model to predict ear moisture content.

Parameters	Ear Moisture Content						
Units	%w.b.						
SEL	0.35						
N	610						
Min	26						
Mean	49						
Max	75						
SD	9.6						
Instruments included	All	1	2	3	1 & 2	2 & 3	1 & 3
SEC	2.8	2.6	2.6	3.8	2.6	2.8	2.6
R²C	0.92	0.94	0.92	0.84	0.93	0.92	0.93
SECV	2.8	2.5	2.8	3.8	2.6	2.9	2.7
R²CV	0.91	0.93	0.91	0.84	0.93	0.91	0.92
Number of terms	8	6	6	6	8	6	8
Segments	10						
Wavelength range/step (nm)	1350–2550/5 ^1^						
Pretreatments	D-2,2,11 ^2^						
Regression method	PLS						

^1^ Average step, ranges from 2.5 at 1350 nm to 8.8 at 2550 nm. ^2^ Savitzky–Golay derivative—smoothing kernel of length 11, quadratic interpolation, second derivative.

**Table 2:** Statistics of validation results derived from using the 2021 ear scans to develop an ear moisture prediction model that was then used to predict scans in 2022.

Parameters	Ear Moisture Content															
Units	%w.b.															
**N**	330															
**Min**	37															
**Mean**	55															
**Max**	80															
**SD**	10															
**Instrument ^1^**	All→All	All→1	All→2	All→3	1→1	1→2	1→3	2→1	2→2	2→3	3→1	3→2	3→3	1&2→3	2&3→1	3&1→2
**R²P**	0.93	0.93	0.93	0.91	0.93	0.91	0.89	0.84	0.93	0.91	0.88	0.88	0.88	0.83	0.87	0.92
**RMSEP**	2.9	1.6	1.6	1.8	2.9	5.2	3.4	5.9	2.8	6.4	4.4	5.0	4.4	4.9	2.7	2.8
**SEP**	2.8	1.6	1.6	1.8	2.8	3.0	3.4	2.8	2.7	3.3	3.7	3.6	3.7	3.2	2.6	2.7
**Bias**	0.52	0.33	0.62	0.61	0.62	4.2	0.28	5.2	0.61	5.4	2.3	3.5	2.3	3.7	2.7	−0.96
**Intercept**	8.6	9.0	7.4	9.4	9.2	10	9.6	14	7.2	17	15	14	15	13	9.7	3.6
**Slope**	0.85	0.84	0.88	0.84	0.84	0.89	0.83	0.84	0.88	0.78	0.77	0.80	0.77	0.83	0.87	0.92

^1^ The first number indicates which instrument(s) were used to collect calibration spectra and the second number indicates which instrument was utilized to collect validation spectra.

## Data Availability

The data presented in this study are available upon request from the corresponding author.
